# Retrospective analysis of somatic mutations and clonal hematopoiesis in astronauts

**DOI:** 10.1038/s42003-022-03777-z

**Published:** 2022-08-17

**Authors:** Agnieszka Brojakowska, Anupreet Kour, Mark Charles Thel, Eunbee Park, Malik Bisserier, Venkata Naga Srikanth Garikipati, Lahouaria Hadri, Paul J. Mills, Kenneth Walsh, David A. Goukassian

**Affiliations:** 1 Cardiovascular Research Institute, Icahn School of Medicine at Mount Sinai, New York, NY USA; 2 Hematovascular Biology Center, Robert M. Berne Cardiovascular Research Center, University of Virginia School of Medicine, Charlottesville, VA USA; 3 Dorothy M. Davis Heart Lung and Research Institute and Department of Emergency Medicine, The Ohio State University Wexner Medical Center, Columbus, OH USA; 4 Center of Excellence for Research and Training in Integrative Health, University of California San Diego, La Jolla, CA USA

**Keywords:** Risk factors, Predictive markers

## Abstract

With planned deep space and commercial spaceflights, gaps remain to address health risks in astronauts. Multiple studies have shown associations between clonal expansion of hematopoietic cells with hematopoietic malignancies and cardiometabolic disease. This expansion of clones in the absence of overt hematopoietic disorders is termed clonal hematopoiesis (CH) of indeterminate potential (CHIP). Using deep, error-corrected, targeted DNA sequencing we assayed for somatic mutations in CH-driver genes in peripheral blood mononuclear cells isolated from de-identified blood samples collected from 14 astronauts who flew Shuttle missions between 1998–2001. We identified 34 nonsynonymous mutations of relatively low variant allele fraction in 17 CH-driver genes, with the most prevalent mutations in *TP53* and *DNMT3A*. The presence of these small clones in the blood of relatively young astronaut cohort warrants further retrospective and prospective investigation of their clinical relevance and potential application in monitoring astronaut’s health.

## Abstract (web summary)

Deep targeted DNA sequencing of peripheral blood mononuclear cells isolated from blood samples from 14 astronauts who flew Shuttle missions between 1998–2001 identifies 34 non-silent mutations, predominantly in TP53 and DNMT3A.

## Introduction

With the advent of NASA’s deep space Gateway and Artemis missions and the expansion of commercial spaceflights, there is an increased need to understand, counteract, and mitigate health risks associated with spaceflight. Specific acute and long-term adverse effects and risks inherent to space missions are defined not only by duration, but also the course of flight, i.e., remaining in low Earth orbit (LEO) where Earth’s magnetic field provides shielding from space radiation or going beyond this protective shield into deep space. Some of these adverse effects include bone-density loss, neurovestibular changes and muscle atrophy. In addition, there are degenerative risks, including space radiation (IR)-induced cancer, cardiovascular and neurodegenerative diseases^1,2^, and all three are priority risk reduction areas identified by NASA’s Human Research Roadmap. Thus, quality health assessments for astronauts that account for an individual’s susceptibility are critical for understanding and addressing risks before, during and after future exploration-type, deep-space missions. Considering baseline genetic and extrinsic variability, the development of tools that permit the assessment of individual genetic susceptibility would improve risk stratification and long-term clinical management.

Somatic mutations occur randomly and in response to various systemic and environmental stressors. While most mutations do not affect cell phenotype, others alter the cell’s fitness, conferring a selective proliferative and survival advantage leading to clonal events in normal tissues. Clonal hematopoiesis (CH) is the expansion of such clones in hematopoietic stem/progenitor cells (HSPCs), resulting in a relatively benign precursor state. The presence of these clonal mutations in the absence of neoplasia at a >2% variant allele frequency (VAF) is designated as CH of indeterminate potential (CHIP)^3^. The accumulation of somatic mutations occurs as a function of aging and can be facilitated by inflammatory alterations in the microenvironment. However, other mechanisms promoting clonal progression remain unclear. Most mutations associated with age-related CHIP occur in epigenetic modifier genes (*DNMT3A, TET2, ASXL1*)^4,5^. In contrast to age-related CHIP, there is an accelerated form of CH that is found in cancer survivors who have been exposed to therapy with genotoxic agents. This is referred to as therapy-associated clonal hematopoiesis (t-CH) and mutations occur predominantly in genes involved in DNA damage response (DDR) pathway (*TP53, PPM1D*)^6^. Studies have associated CHIP with an increased risk of hematological and solid malignancies, cardiovascular disease (CVD), cardiometabolic and neurodegenerative disease, and overall higher mortality^4,5,7^. Notably, the size of mutant clones is associated with increased risk; however, additional factors such as comorbidities or cell-extrinsic factors, are likely to impact the degree of risk for such outcomes. Additionally, experimental studies have shown that CH can contribute to chronic diseases by promoting inflammation^8–10^.

Spaceflights are associated with exposure to various stressors, including IR, microgravity, and other harmful space environmental factors. NASA’s Twin study reported hematopoietic mutations in *DNMT3A* and *TET2*, consistent with the presence of CHIP^11^. While the Twin studies employed conventional next generation, whole genome sequencing, the current study employed deep, error-corrected DNA sequencing to detect somatic mutations in CH-driver genes using de-identified blood samples collected from 14 astronauts who flew Space Transportation System (STS), aka Space Shuttle, missions between 1998–2001. We identified 34 non-silent mutations, predominantly in *TP53* and *DNMT3A*, consistent with the presence of CH in this relatively young astronaut cohort.

## Results

**Fig. 1:**
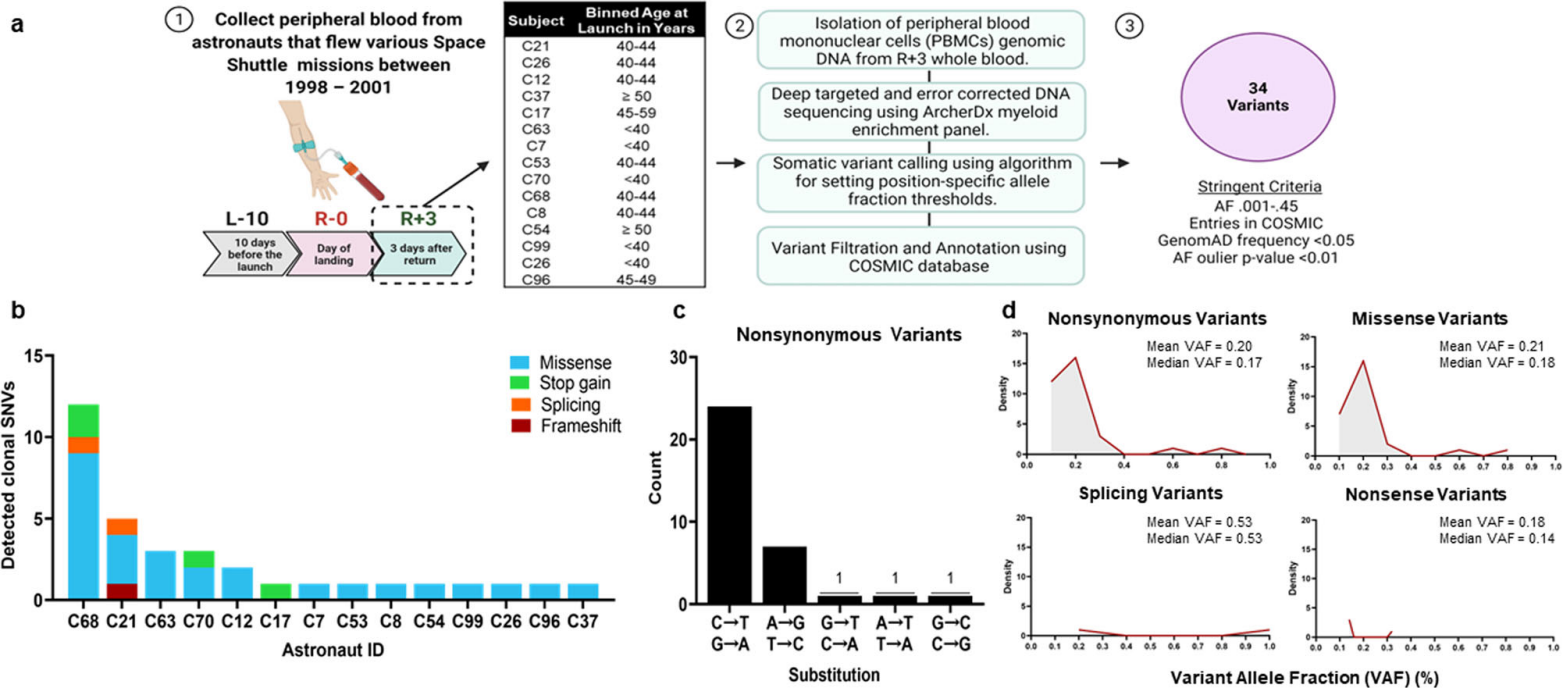
Characteristics of Clonal Hematopoiesis (CH) Mutations. **a** We identified somatic mutations in known clonal hematopoiesis of indeterminate potential (CHIP) driver genes using peripheral blood mononuclear cells isolated from 14 astronauts who flew short space Shuttle missions lasting a median of 12 days between 1998–2001. Created with BioRender.com. **b** Number of somatic nonsynonymous single nucleotide variants (SNVs) in CHIP-driver genes harbored per subject. **c** Rates of different substitution types observed in clonal SNVs. Only one guanine to thymine transition was observed. **d** Density of mutations by VAF for each mutation type.

We obtained de-identified whole blood samples from 14 astronauts who flew relatively short Space Shuttle missions (median 12 days) between 1998–2001. These samples were stored at −80 °C for ~20 years. Blood samples were collected 10 days before flight, the day of landing, and 3 days after landing^12^. However, for this specific study, only samples from 3 days after landing (R + 3) were collected as buffy coats (peripheral blood mononuclear cells - PBMCs). The median age of the Shuttle mission crew (including individuals who did not donate blood) at sample collection was approximately 42 years (IQR = 39–45) (Fig. 1a). Six of the 14 (43%) astronauts were on their first mission at time of sample collection. Approximately 85% of crew members were male during missions conducted between 1998–2001. An average of two extravehicular activities (EVAs) occurred per Shuttle mission, with two astronauts participating per EVA (an average of four astronauts per mission). Per the Lifetime Surveillance of Astronaut Health (LSAH) at NASA Johnson Space Center, the astronauts who participated in the following study did not have any prior exposures to chemotherapy or radiation therapy. To detect somatic mutations in CH-driver genes in PBMCs, we performed deep, error-corrected DNA sequencing using a myeloid panel to enrich 37 candidates frequently mutated in myeloid malignancies. Mutational analysis of PBMCs identified 34 somatic nonsynonymous single nucleotide variants (SNVs) in 17 genes at 0.10–0.95% (median 0.18%) VAF (Fig. 1a, d). Five out of 14 astronauts (36%) harbored two or more mutations. Subjects C68 and C21 harbored the largest number of clonal SNVs, 12 and 5, respectively (Fig. 1b). We detected 27 missense SNVs (median VAF 0.18%), 4 nonsense SNVs (median VAF 0.14%), 2 splicing SNVs (median VAF 0.53%), and 1 frameshift SNV (Fig. 1b, d). The most common exonic substitution was cytosine to thymine (C → T) transition, followed by guanine to adenine (G → A) transition (Fig. 1c), which is considered a mutational signature of aging^13^ and is common in variants associated with CHIP.

**Fig. 2:**
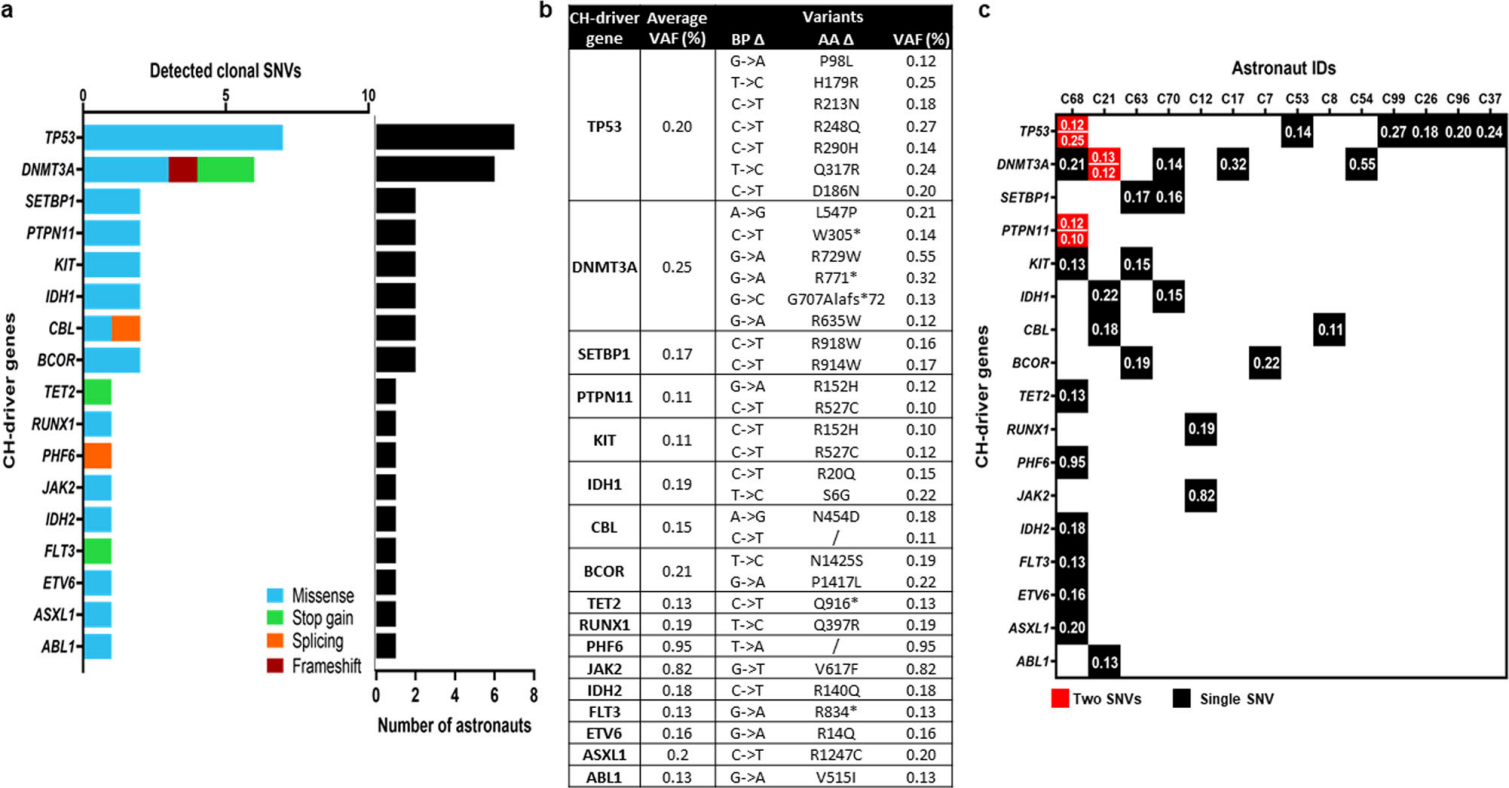
Clonal Hematopoiesis (CH) Profile of Astronauts. **a** Mutational profile of 17 known mutated CHIP-driver genes and number of astronauts with mutations in each gene. **b** Table with average VAF, noted nucleotide (BPΔ) and amino acid (AAΔ) changes for each nonsynonymous mutation. Asterix (*) refers to terminated sequencing for stop gain mutations while backslash (/) corresponds to splicing mutations where AAΔ is not applicable. **c** Mutation plot for each individual is represented as columns. Each rectangle represents number of mutations identified in each gene, with black being 1 mutation and red 2 mutations, along with individual VAF (%) for each variant.

**Fig. 3:**
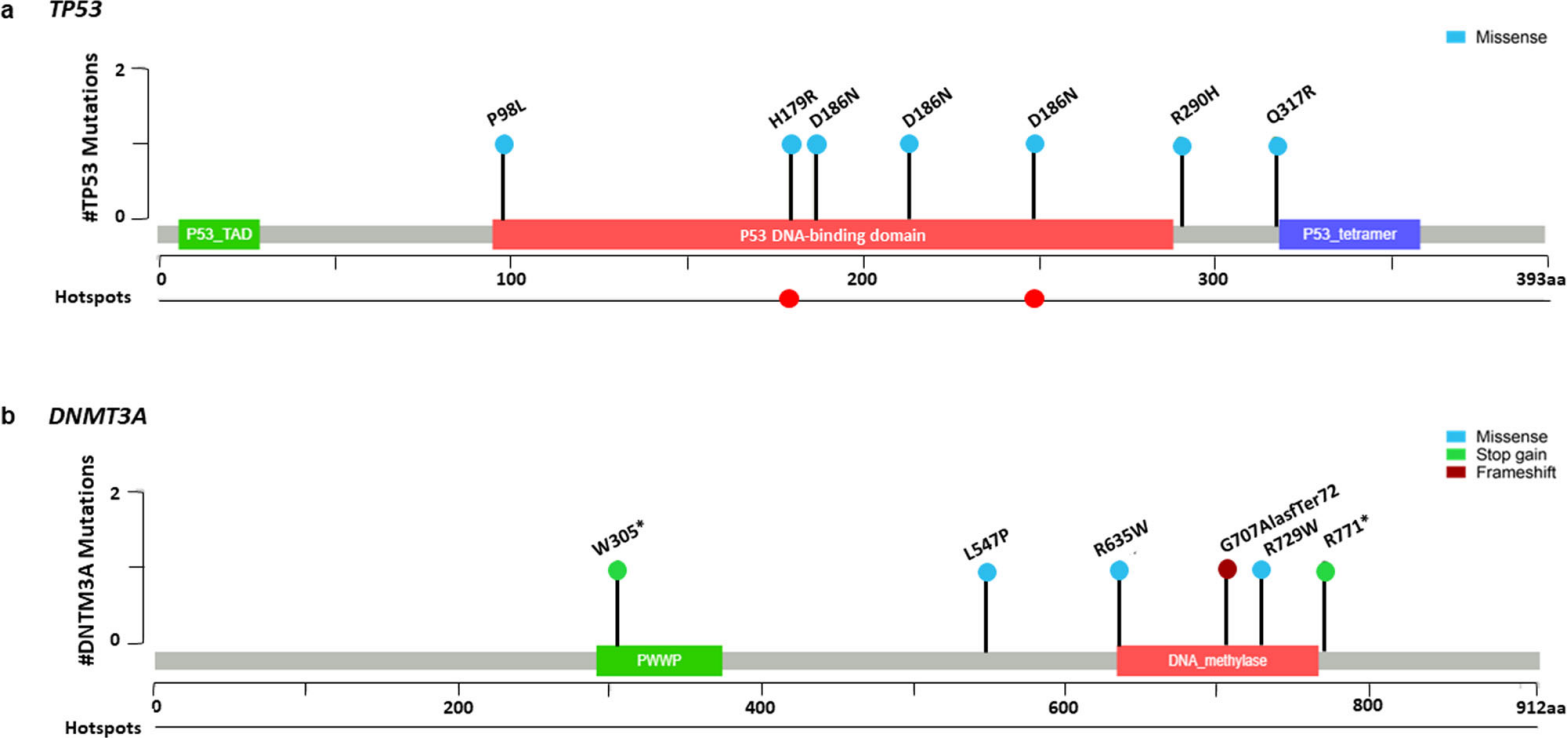
Profile of nonsynonymous mutations for TP53 and DNMT3A. Mapping of nonsynonymous mutations in (**a**) TP53 and (**b**) DNMT3A through Loliplot visualization. Clinically relevant hotspots are identified (red dots). Asterix (*) in mutations corresponds to sequence termination in stop gain mutations. *P53_TAD: p53 transactivation domain; PWWP: Pro-Trp-Trp_Pro domain*.

We detected variants in 17 of the 37 genes represented in the panel. The most commonly mutated gene was *TP53* (7 variants) followed by *DNTM3A* (6 variants) accounting for 38% of mutations detected (Fig. 2a). One subject, C12, harbored the *JAK2* V617F mutation (VAF 0.82%) (Fig. 2b, c), a clinically relevant variant identified in myeloproliferative neoplasms (MPNs) that has also been associated with ~12-fold increased risk of CVD and thrombosis risk^5,14–17^. Subject C68 had a single *PHF6* SNV with a VAF of 0.95% (Fig. 2b, c), representing the largest variant clone detected by this analysis. While the pathogenicity of this mutation is unclear, *PHF6* variants are frequently detected in patients with hematopoietic malignancies^18^. Multiple mutations in *TP53* and *PTPN11* were also identified in subject C68, and four other subjects harbored co-mutations in other CH-driver genes (Fig. 2c). All *TP53* SNVs missense mutations occurred in the DNA binding domain. Two mutations were located in the *TP53* mutation hotspots: Arg248Gln (VAF 0.27%) and His179Arg (VAF 0.25%) (Fig. 3a), which are implicated in various cancers^19–21^. Six out of 14 astronauts (43%) had at least 1 *TP53* SNV (average VAF 0.20%), of which subject C68 had two missense mutations (Fig. 2c). In comparison, 5/14 (36%) of astronauts had at least one *DNMT3A* SNV, which were predominantly missense mutations in the methyltransferase domain, thus likely impacting enzyme function, or nonsense mutations (Arg771Ter, Trp305Ter) that had been identified in patients with aplastic anemia and angioimmunoblastic T Cell lymphoma^22,23^ (Fig. 3b). Subject C21 had two *DNMT3A* SNVs with 1 missense and 1 frameshift variant (Fig. 2c).

## Discussion

The advancement of error-corrected DNA sequencing technology permits the assessment of somatic mutations (at lower VAF) in CH-driver genes associated with increased risk of hematologic and non-hematologic cancers as well as cardiovascular diseases^24^. This study used PBMCs collected ~20 years ago from 14 relatively young astronauts who flew space Shuttle missions between 1998–2001 to identify, retrospectively, somatic mutations in CH-driver genes.

We identified 34 nonsynonymous SNVs in 17 known CH-driver genes, of which *TP53* and *DNMT3A* were the most frequent. Notably, clone size was small, ranging from 0.10% to 0.95% VAF, and thus did not achieve the technical threshold to be considered as CHIP. In contrast, a population-based CH study of middle-aged participants (median age 58 years, range 19 to 108) showed that the most frequent variants occur in epigenetic modifier genes *DNMT3A*, followed by *TET2*, *ASXL1*, and *TP53*^4^. However, the DDR gene *TP53* was the most frequently mutated in this astronaut cohort (median age 44 years, range 37–67), reflecting a potential difference compared to the civilian population^4,5^. Somatic *TP53* mutations are uncommon in patients without a history of cancer therapy^25^. *TP53* encodes tumor suppressor p53 that is induced by various stress stimuli, including UV IR, DNA double-stranded breaks, and oxidative stress. *TP53* coordinates the transcription of genes involved in DNA damage repair, apoptosis, growth arrest, or senescence, and the dysregulation of p53 poses a risk for developing cancers, CVD, and other metabolic disorders^26,27^. While the clones identified in this astronaut cohort are relatively small, there is a possibility for increased clonal instability with aging that may be facilitated by co-mutations in other driver genes. Indeed, 5/14 astronauts harbored mutations in at least two CH-driver genes, and subject C68 had variants in the four mutated genes. While we cannot assess the pathogenic risk and the clinical value for each small clone mutation, it provides a rationale for longitudinal studies to determine which clones are at high risk of transformation and then to develop strategies to inhibit their expansion at early stages.

Due to the lack of longitudinal samples and small sample size, conclusions regarding the implications of observed lesions remain limited, and further studies are required to assess the penetrance of these clones. Van Egeren et al. showed single HSPCs acquired *JAK2-*V617F mutations decades before the development of MPNs, with clonal growth and/or extinction often fluctuating years after mutation acquisition^28^. While the initial occurrence of these CH lesions within our astronaut cohort cannot be ascertained, the spaceflight environment introduces considerable physiological stress, which could temporally alter clonal dynamics. Our team recently showed plasma cell-free mitochondrial DNA (cf-mtDNA) and 8-OHdG levels were elevated in these astronauts 3 days post-flight (R + 3) and found inflammatory (*TNFα, IL-1α, IL-1β*), oxidative stress (*SOD1, GPX1, NOX4, SERPINE1, HMOX1*), and DNA damage (*OGG1, PARP-1*) markers were significantly upregulated at R + 3 compared to baseline (10 days before flight) in isolated PBMCs^29^, suggesting spaceflight contributes to significant physiological stress, which may be an impetus for clonal selection and expansion. While we cannot ascertain which isolated space environment factor may pose considerable risks for CH development, considering the clonal landscape in the following astronaut cohort bares closer resemblance to t-CH, it is possible space IR may be a primary driver of clonal selection. Space IR comprises of high charge and energy (HZE) ions with a high linear energy transfer (LET) and provides different energy deposition patterns in cells creating densely ionizing tracks that generate clustered DNA damage compared to low LET terrestrial IR. Prior studies examining the risk of cataracts following space IR exposure during Shuttle missions noted several thousand high-LET particles (LET > 30 keV/µm) pass through the lens with an average scaled mission lens dose of 73.5 mSv (0.07 Gy) for missions of 10 to 14-day duration^30,31^. Additionally, astronauts within the studied cohort embarked on EVAs during their Shuttle mission which also modifies degree of stressor exposure. While most EVAs are scheduled around solar activity, most galactic cosmic rays (GCRs) are unpredictable, and spacesuit radiation shielding alone may not be enough to mitigate radiation exposure and its associated risks^32^. Considering cancer patients receiving external beam radiation therapy exhibit pronounced CH mutations in DDR genes (*TP53, CHEK2, PPM1D*) along with faster clone growth following cumulative exposure^33^, it can be postulated that exposure to space IR may alter the fitness landscape of observed CH mutations and warrants further investigation. Additionally, the effect of sex on CH dynamics should be considered as approximately 85% of crew members in Shuttle Missions from 1998–2001 were male. Prior studies have shown the male sex acquired more somatic mutations with higher allele burdens in known CH-drivers such as *JAK2, ASXL1, SRSF2, U2AF1*, and *IDH1/2* and were associated with overall poorer clinical outcomes^34,35^. Thus, more comprehensive studies are required to ascertain whether sex modifies space environment-induced CH risk using a more diverse cohort.

Overall, further longitudinal studies are required to characterize CH and somatic mutational profiles in the context of space flight-associated stressors and their associated clinical impact. To date, there is no evidence of relevant CVD, cancer, or neurodegenerative diagnoses associated with this given astronaut cohort (current median age 62.5 years (IQR 60–67)). The lack of longitudinal samples from these same astronauts limits the assessment of clone stability, pathogenic potential, and prognostic value. Studies of CH in astronauts would provide a new tool for assessing individual risk and potentially optimize long-term health monitoring. Additional analyses would also offer an opportunity to address the paucity of longitudinal CH studies using a unique cohort of physically fit individuals and provide more insights into factors that influence CH in the general population. Thus, this study serves to address the feasibility of using bio-banked astronaut samples and demonstrate the importance of collaborations between NASA’s Human Research Program, Translational Research Institute for Space Health, Space Biology Program, NASA’s clinical support teams and corresponding data and biorepository branches, and NASA IRB to facilitate retrospective and prospective longitudinal studies by increasing sample availability (while protecting health information (PHI) and maintaining HIPAA regulations). This integration of clinical data could optimize the value of CHIP studies for the assessment of lifetime risks before and after spaceflight.

## Methods

### Subjects and samples

We obtained de-identified whole blood samples from 14 astronauts who flew relatively short (median 12 day long) space Shuttle missions, between 1998–2001. Identifying information related to these samples is limited to the crew’s binned age in order to ensure privacy of involved personnel (Fig. 1a). De-identified aliquots of 14 peripheral blood mononuclear cell (PBMC) samples, isolated from blood samples 3 days after return (R + 3; during original study in 1998–2001; note, the buffy coats to obtain PBMCs were collected only at this time point), were prepared and shipped overnight to the University of Virginia School of Medicine for detection of somatic mutations involved in known CHIP-driver genes (Fig. 1a). Given that samples are de-identified and targeted DNA sequencing would not permit individual identification, NASA’s Institutional Review Board (IRB) deemed this study non-human research (STUDY00000075) and the Icahn School of Medicine at Mount Sinai’s IRB deemed this study as exempt (HSM19-00367). Blood samples were collected during a NASA flight study; astronauts provided written informed consent to participate in that study and have blood stored and used for residual analyses^12^.

### Sample preparation and targeted DNA sequencing

Genomic DNA was isolated from PBMCs from each sample using the QIAamp DNA Blood Mini kit (Cat #51104). DNA quality was assessed using the PreSeq QC kit (Cat # SA0597 and SA0598) provided by ArcherDX (now Invitae) using real-time polymerase chain reaction (RT-PCR). ArcherDX VariantPlex core myeloid kit with 37 genes panel (Supplementary Table 1) was used to perform target specific PCR using Anchored Multiplex PCR (AMP™) chemistry to add specific Molecular Barcodes (MBC) and indexes. DNA libraries were prepared using Archer Library Preparation Reagents for Illumina (Cat #AB0101) by following the manufacturer’s protocol. Library sequencing was performed using Illumina NextSeq platform with high output kits as per manufacturer guidelines to generate 150 bp; paired end data. Sequencing was performed to 15000X average coverage. NGS data was analyzed using the Archer Analysis software (https://analysis.archerdx.com/) including the following steps. Raw paired end FASTQ files were subjected to read cleaning and adaptor trimming to remove adapter sequences and low-quality portions of reads at 5’ and 3’ ends. Then unique reads / read pairs were grouped together into “molecular bins”, according to molecular barcode and index sequences. Bins were subjected to deduplication and error correction to create a consensus read for each bin. A combination of bowtie2 and BWA-MEM was used to map reads against the reference human genome (hg19/GCRh37). Generated variants were annotated with Ensembl Variant Effect Predictor.

Variants (SNP/ InDel) generated with this method were compared with a normal dataset using Archer’s analysis pipeline to distinguish noise from a true call. The normal dataset was created with sequencing data from seven young, healthy individuals. Based on the frequency at which a variant is observed in the normal dataset, the noise distribution at each variant call was calculated. Then the probability was calculated for the observed variant AF that could have been generated by noise given that noise distribution. Low normal data set AF outlier *p* value indicated that a variant was unlikely to have been generated by noise.

A maximum VAF threshold of 0.40 was used to exclude potential germline mutation from variant calling. Below this threshold, variants were further filtered if (1) AF outlier *p* ≤ 0.01; (2) GenomeAD frequency ≤ 0.05; (3) no previous report of the variant identified in Catalog of Somatic Mutations in Cancer or ClinVar; (4) duplicate variant per sample. The final data set is available in Supplementary Data 1.

### Statistics and reproducibility

Due to limited sample availability, there is no replicate for the sequencing. Strict criteria for sequencing data quality check were applied to each sample. All the samples passed the QC status that is determined by the Invitae (Archer) data analysis pipeline (https://analysis.archerdx.com/). Statistical evaluation for sequencing data from each sample is presented in Supplementary Data 2. As stated above, only true somatic variants were selected by applying the strict filtering criteria of AF outlier *p* ≤ 0.01, present in COSMIC and GenomeAD AF ≤ 0.05. Binomial distribution was used to model the noise at each position from the sequencing data of the normal data set to generate the AF outlier *p*-value. The background noise model was established using these steps: (i) Identified base changes with unusually high AF and exclude them using Inter Quantile Range (IQR) filtering. (ii) For remaining variants: (a) Background noise = Sum of all AOs (Alternate observations) (across all samples)/Sum of depth (across all samples) at a given position, on per base change basis. If AO = 0, background noise was assumed to be 1/Sum of the depth (across all samples). (b) The background noise calculated in (A) became the expected rate of AO observations per unique molecule at a given position. This rate was used as the “*p*” term in a binomial distribution. (c) For a given variant, analysis can calculate a *p* value which expresses the probability that the noise model for that variant would generate the observed AF or greater. This assumes a binomial distribution with *p* = background noise and *n* = depth of coverage for that variant, which computes the probability of the null hypothesis (*p*-value). The null hypothesis was that the number of alternate observations seen are due to the background error that was estimated. (d) Any variant that had a low *p*-value was considered a significant variant. (The recommended default *p*-value cutoff, or alpha, is 0.01, but this can be adjusted based on tolerance for false positives and false negatives).

### Reporting summary

Further information on research design is available in the Nature Research Reporting Summary linked to this article.

## Supplementary information

Supplementary Information

Description of Additional Supplementary Data

Supplementary Data 1

Supplementary Data 2

Reporting Summary

## Data Availability

All data derived from analysis of clinical sequencing data (CH mutations) for all astronauts necessary to replicate the findings in the article are available within the main text and supplementary materials. The raw sequencing data for the astronaut cohorts are protected and are not broadly available due to privacy laws. The LSDA provides an appropriate process for archival of experimental data and dissemination, which complies with policies to govern sensitive data in accordance with NASA Human Research Program and Johnson Space Center (JSC) Institutional Review Board direction. Raw data elements may be requested from david.goukassian@mssm.edu with appropriate institutional approvals.
