# Author Correction: Retrospective analysis of somatic mutations and clonal hematopoiesis in astronauts

**DOI:** 10.1038/s42003-022-04071-8

**Published:** 2022-10-10

**Authors:** Agnieszka Brojakowska, Anupreet Kour, Mark Charles Thel, Eunbee Park, Malik Bisserier, Venkata Naga Srikanth Garikipati, Lahouaria Hadri, Paul J. Mills, Kenneth Walsh, David A. Goukassian

**Affiliations:** 1 Cardiovascular Research Institute, Icahn School of Medicine at Mount Sinai, New York, NY USA; 2 Hematovascular Biology Center, Robert M. Berne Cardiovascular Research Center, University of Virginia School of Medicine, Charlottesville, VA USA; 3 Dorothy M. Davis Heart Lung and Research Institute and Department of Emergency Medicine, The Ohio State University Wexner Medical Center, Columbus, OH USA; 4 Center of Excellence for Research and Training in Integrative Health, University of California San Diego, La Jolla, CA USA

**Keywords:** Risk factors, Predictive markers

Correction to: *Communications Biology* 10.1038/s42003-022-03777-z, published online 17 August 2022.

In the Acknowledgements section, “This work was supported by the Translational Research Institute for Space Health award FIP0005 (to Dr. Goukassian)” should have read “This work is supported by the Translational Research Institute for Space Health through NASA Cooperative Agreement NNX16AO69A – FIP0005”.

The original article has been corrected.

