# Supplementary material for: Retrospective analysis of somatic mutations and clonal hematopoiesis in astronauts
Source: Commun Biol. 2022 Aug 17;5:828. doi: 10.1038/s42003-022-03777-z (PMC9385668; doi:10.1038/s42003-022-03777-z)
Supplement: Supplementary file 1 — Supplementary Information [file 42003_2022_3777_MOESM1_ESM.pdf]

Supplementary Table 1. Gene list in VariantPlex Core Myeloid Panel

ABL1	CALR	ETNK1	GATA1	KIIT	PHF6	SRSF2	WT1
ANKRD26	CBL	ETV6	GATA2	KRAS	PTPN11	STAG2	ZRSR2
ASXL1	CEBPA	EZH2	IDH1	MPL	RUNX1	TET2	
BCOR	CSF3R	FLT3	IDH2	NPM1	SETBP1	TP53	
BRAF	DNMT3A	DDX41	JAK2	NRAS	SF3B1	U2AF1	
