# Supplementary material for: Retrospective analysis of somatic mutations and clonal hematopoiesis in astronauts
Source: Commun Biol. 2022 Aug 17;5:828. doi: 10.1038/s42003-022-03777-z (PMC9385668; doi:10.1038/s42003-022-03777-z)
Supplement: Supplementary file 2 — Description of Additional Supplementary Data [file 42003_2022_3777_MOESM2_ESM.pdf]

Description of Additional Supplementary Files

File name: Supplementary Data 1
Description: Source data of somatic variants detected and used for graphs in the manuscript

File name: Supplementary Data 2
Description: Statistical evaluation for sequencing data from each sample
