# Supplementary material for: Retrospective analysis of somatic mutations and clonal hematopoiesis in astronauts
Source: Commun Biol. 2022 Aug 17;5:828. doi: 10.1038/s42003-022-03777-z (PMC9385668; doi:10.1038/s42003-022-03777-z)
Supplement: Supplementary file 5 — Reporting Summary [file 42003_2022_3777_MOESM5_ESM.pdf]

nature research

Corresponding author(s): David Goukassian

Last updated by author(s): 07/07/2022

Reporting Summary

Nature Research wishes to improve the reproducibility of the work that we publish. This form provides structure for consistency and transparency in reporting. For further information on Nature Research policies, see our Editorial Policies and the Editorial Policy Checklist.

Statistics

For all statistical analyses, confirm that the following items are present in the figure legend, table legend, main text, or Methods section.

n/a	Confirmed	
☐	☒	The exact sample size (*n*) for each experimental group/condition, given as a discrete number and unit of measurement
☐	☒	A statement on whether measurements were taken from distinct samples or whether the same sample was measured repeatedly
☐	☒	The statistical test(s) used AND whether they are one- or two-sided<br>*Only common tests should be described solely by name; describe more complex techniques in the Methods section.*
☐	☒	A description of all covariates tested
☐	☒	A description of any assumptions or corrections, such as tests of normality and adjustment for multiple comparisons
☐	☒	A full description of the statistical parameters including central tendency (e.g. means) or other basic estimates (e.g. regression coefficient) AND variation (e.g. standard deviation) or associated estimates of uncertainty (e.g. confidence intervals)
☒	☐	For null hypothesis testing, the test statistic (e.g. *F*, *t*, *r*) with confidence intervals, effect sizes, degrees of freedom and *P* value noted<br>*Give P values as exact values whenever suitable.*
☒	☐	For Bayesian analysis, information on the choice of priors and Markov chain Monte Carlo settings
☒	☐	For hierarchical and complex designs, identification of the appropriate level for tests and full reporting of outcomes
☒	☐	Estimates of effect sizes (e.g. Cohen's *d*, Pearson's *r*), indicating how they were calculated

Our web collection on statistics for biologists contains articles on many of the points above.

Software and code

Policy information about availability of computer code

Data collection	Archer Invitae Bioinformatics pipeline was used for data collection and data analysis.
Data analysis	NGS data was analyzed using the Archer (Invitae) Analysis software (https://analysis.archerdx.com/).

For manuscripts utilizing custom algorithms or software that are central to the research but not yet described in published literature, software must be made available to editors and reviewers. We strongly encourage code deposition in a community repository (e.g. GitHub). See the Nature Research guidelines for submitting code & software for further information.

Data

Policy information about availability of data

All manuscripts must include a data availability statement. This statement should provide the following information, where applicable:

- Accession codes, unique identifiers, or web links for publicly available datasets
- A list of figures that have associated raw data
- A description of any restrictions on data availability

All data derived from analysis of clinical sequencing data (CH mutations) for all astronauts necessary to replicate the findings in the article are available within the main text and supplementary materials. The raw sequencing data for the astronaut cohorts are protected and are not broadly available due to privacy laws. The LSDA provides an appropriate process for archival of experimental data and dissemination, which complies with policies to govern sensitive data in accordance with NASA Human Research Program and Johnson Space Center (JSC) Institutional Review Board direction. Raw data elements may be requested from david.goukassian@mssm.edu with appropriate institutional approvals.

Field-specific reporting

Please select the one below that is the best fit for your research. If you are not sure, read the appropriate sections before making your selection.

☒ Life sciences ☐ Behavioural & social sciences ☐ Ecological, evolutionary & environmental sciences

For a reference copy of the document with all sections, see nature.com/documents/nr-reporting-summary-flat.pdf

Life sciences study design

All studies must disclose on these points even when the disclosure is negative.

Sample size	Blood samples were collected from astronauts who participated in a NASA flight study conducted by our collaborator Dr. Paul Mills (PMID 12417448). Participants provided written informed consent to participate in that study and have blood stored and used for residual analyses. Our study focuses on 14 astronauts who flew aboard eight different US Space Shuttle flights between 1997 and 2001. Buffy coats used in this study were isolated only from samples collected within 4 days post landing (R+3).<br><br>No sample size calculation was performed for the following study. Samples were chosen based on availability of “buffy coats” for 14 astronauts from the original protocol performed by Dr. Paul Mills. This study aimed to identify whether spaceflight may induce somatic mutations in 37 genes (from ArcherDx Myeloid Enrichment Panel) that are known clonal hematopoeisis (CH) “driver” genes and to demonstrate the feasibility of using retrospectively collected samples for such work.
Data exclusions	Only true somatic variants were selected by applying the strict filtering criteria of AF outlier p value <= .01, present in COSMIC and gnomAD AF <=.05.<br>Binomial distribution is used to model the noise at each position from the sequencing data of the normal data set to generate the AF outlier p-value. The background noise model is established using these steps:<br>1) Identify base changes with unusually high AF and exclude them using Inter Quantile Range (IQR) filtering.<br>2) For remaining variants:<br>a. Background noise = Sum of all AOs (Alternate observations) (across all samples) / Sum of depth (across all samples) at a given position, on per base change basis. If AO = 0, background noise is assumed to be 1 / Sum of the depth (across all samples).<br>b. The background noise calculated in (A) becomes the expected rate of AO observations per unique molecule at a given position. This rate is used as the "p" term in a binomial distribution.<br>c. For a given variant, analysis can calculate a p value which expresses the probability that the noise model for that variant would generate the observed AF or greater. This assumes a binomial distribution with p = background noise and n = depth of coverage for that variant, compute the probability of the null hypothesis (p-value). The null hypothesis is that the number of alternate observations seen are due to the background error that was estimated.<br>d. Any variant that has a low p-value will be considered a significant variant. (The recommended default p-value cutoff, or alpha, is .01, but this can be adjusted based on tolerance for false positives and false negatives).
Replication	Due to availability of the samples and the cost of sequencing there is no replicate for the sequencing. Strict criteria for sequencing data quality check were applied to each sample. All the samples passed the QC status that is determined by the Invitae (Archer) data analysis pipeline. Statistical evaluation for sequencing data from each sample is presented in Supplementary Data 2. Low quality reads were removed during molecular binning. Extra stringency to the analysis was obtained by analysis setting for different parameters as suggested by Invitae’s (Archer’s) guidelines. Description for analysis setting guidelines is presented in Supplementary Data 2.
Randomization	This is a feasibility study to identify if CH is present in young astronauts and whether this may be a tool to help identify individual susceptibility to possible adverse health effects associated with space flight. In addition, this pilot study serves to address the feasibility of using bio-banked astronaut samples and demonstrate the importance of collaborations between NASA's Human Research Program, Translational Research Institute for Space Health, Science Mission Directorate/Space Biology Program, NASA clinical branches (Medical Operations, Flight Surgeons), Lifetime Surveillance of Astronauts Health (LSAH), NASA’s Life Sciences Data Archive (LSDA) and NASA IRB to facilitate retrospective and prospective longitudinal studies by increasing sample availability (while protecting health information (PHI) and maintaining HIPAA regulations).<br><br>Thus, our primary focus was to assess the somatic mutational profiles of peripheral blood mononuclear cells (PBMCs) isolated from this astronaut cohort. Given the limited sample size of this cross-sectional study and limitations in access to additional demographic and clinical information, the ability to allocate samples into groups stratified by other covariates (gender, the number of space flights flown, etc.) was limited.
Blinding	Blood samples were collected from astronauts who participated in a NASA flight study conducted by our collaborator Dr. Paul Mills under the approval of the Johnson Space Center Institutional Review Board (PMID 12417448). Aliquots of buffy coat samples were transferred to Dr. David Goukassian’s laboratory from Dr. Paul Mill’s laboratory through signing the official MTA. Dr. Goukassian does not have and will not have the code key to the samples that were assigned at the time of the sample collection. All samples were further de-identified once received, with new random numbers. Information regarding these samples is limited to the date of sample collection. Initially at the time of collection all samples were coded with random 4 digit numbers. For this publication we further coded the samples using a random code generator.

Reporting for specific materials, systems and methods

We require information from authors about some types of materials, experimental systems and methods used in many studies. Here, indicate whether each material, system or method listed is relevant to your study. If you are not sure if a list item applies to your research, read the appropriate section before selecting a response.

Materials & experimental systems

n/a	Involved in the study	
☒	☐	Antibodies
☒	☐	Eukaryotic cell lines
☒	☐	Palaeontology and archaeology
☒	☐	Animals and other organisms
☐	☒	Human research participants
☒	☐	Clinical data
☒	☐	Dual use research of concern

Methods

n/a	Involved in the study	
☒	☐	ChIP-seq
☒	☐	Flow cytometry
☒	☐	MRI-based neuroimaging

Human research participants

Policy information about studies involving human research participants

Population characteristics	Blood samples were collected from astronauts who participated in a NASA flight study conducted by our collaborator Dr. Paul Mills under the approval of the Johnson Space Center Institutional Review Board (PMID 12417448). Written informed consents were obtained for all astronauts. All samples were de-identified and were given a random 4 digit code. Drs. Janice Meck and Paul Mills, Principal Investigators on this initial project, published three studies using these samples, where the detailed methodology is described. Aliquots of buffy coat samples were transferred to Dr. David Goukassian's laboratory from Dr. Paul Mill's laboratory through signing the official MTA. Dr. Goukassian does not have and will not have the code key to the samples that were assigned at the time of the sample collection. All samples were further de-identified once received, with new random numbers using a random number generator.<br><br>Our study focuses on 14 astronauts who flew aboard 8 different US Space Shuttle flights between 1998 and 2001. The median duration of these 8 missions was 12 days. Buffy coats were isolated only from samples collected within 4 days post landing (R+3).<br><br>All samples were additionally de-identified before being received by our lab for processing; thus, no specific demographic information (exact age, gender, number of flights flow, etc.) nor clinical data is available. Along with the help from the Lifetime Surveillance of Astronaut Health (LSAH) at NASA Johnson Space Center, we were able to identify the median age of the Shuttle mission crew (including individuals who did not donate blood) at sample collection was approximately 42 years (IQR = 39-45). The astronauts who participated in the following studies did not have any prior exposures to chemotherapy or radiation therapy. To date, there is no evidence of relevant CVD, cancer, or neurodegenerative diagnoses associated with this given astronaut cohort. Additionally, approximately 85% of astronauts were male during the period of the Shuttle missions assessed (1998-2001). Six of 14 (43%) of studied astronauts were on their first flight, and an average of two extravehicular activities (EVAs) were completed per mission with two astronauts participate per EVA (avg. 4 astronauts per mission).
Recruitment	Participants were recruited per the original protocol conducted by Dr. Paul Mills as approved by the Johnson Space Center Institutional Review Board. As samples used were de-identified, the following study was deemed non-human research by NASA IRB as no additional information regarding recruitment could be obtained in order to protect private health information (PHI).
Ethics oversight	Given that samples are de-identified and targeted DNA sequencing would not permit individual identification, NASA's Institutional Review Board (IRB) deemed this study non-human research (STUDY00000075) and the Icahn School of Medicine at Mount Sinai's IRB deemed this study as exempt (HSM19-00367).

Note that full information on the approval of the study protocol must also be provided in the manuscript.
